# A Case of Nystagmus

**Published:** 1911-12-23

**Authors:** 


### A Case of Nystagmus.

The fact that a workman may recover even where he has not given the statutory notice of accident is emphasised in the case of Moore *v.* Naval Colliery Company in the Court of Appeal on October 18.

The County Court Judge had refused to award claimant compensation on account of suffering from nystagmus on the ground that he had not made his claim within the statutory period.

The workman found that he was suffering from miner's nystagmus. One of the curious things about nystagmus was that if a man abstained for a time from working underground the disease was likely to leave him.

The man consulted his doctor but unfortunately a strike broke out in his district which lasted a considerable time, and he was not able to work any longer because the colliery in which he was employed came to a standstill. The man thought that the nystagmus would pass away during the strike, and he did not do anything at the time, but finding that the nystagmus did not improve he consulted Dr. Cresswell, of Cardiff, who suggested that he should see the certifying surgeon, who should certify that he was suffering from nystagmus. The certifying surgeon inserted a date but the employers were not prejudiced by that. If there had been a failure he asked their lordships to say that such failure had been made by mistake and that it was provided for by the terms of the Act.

The Master of the Rolls giving judgment said in his opinion there had been reasonable cause for the delay in registering the application, and he thought the learned judge was wrong in the decision at which he had arrived; the appeal would, therefore, be allowed, and the case sent back to the County Court judge.

The Lords Justices concurred and the appeal was therefore allowed.

